# Supplementary material for: Does amyloid fibril nucleation occur at surfaces only?
Source: Biophys J. 2025 Nov 6;125(1):29–34. doi: 10.1016/j.bpj.2025.11.002 (PMC12821009; doi:10.1016/j.bpj.2025.11.002)
Supplement: Document S1. Figures S1–S5 [file mmc1.pdf]

Biophysical Journal, Volume *125*

Supplemental information

Does amyloid fibril nucleation occur at surfaces only?

Jon Pallbo, Sara Linse, and Ulf Olsson

Supporting information for:

Does amyloid fibril nucleation occur at surfaces only?

Jon Pallbo[1]*, Sara Linse[2], and Ulf Olsson[1]

[1]Physical Chemistry, Lund University, P.O. Box 124, 221 00 Lund, Sweden

[2]Biochemistry and Structural Biology, Lund University, P.O. Box 124, 221 00 Lund, Sweden

*jon.pallbo_arvidsson@fkem1.lu.se

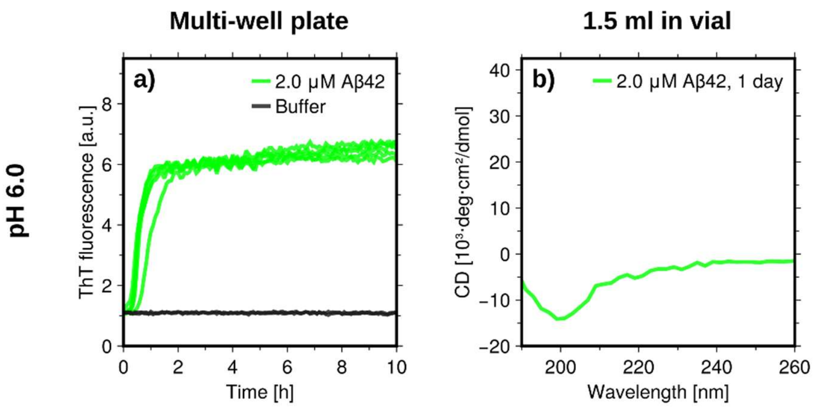

Figure S1. Comparison between amyloid formation in **a)** a multi-well plate (Corning 3881, 96-well half area) and **b)** a vial (borosilicate glass, 13 mm inner diameter). The same Aβ42 monomer solution (2.0 µM) was used to prepare all samples. The multi-well plate samples had aggregated within 2 hours, whereas no sign of aggregation was seen for the sample in the vial after one day of quiescent incubation. The buffer was the same as for the other experiments (at pH 6.0) with the addition of 9 µM Thioflavin T (ThT) in all samples.

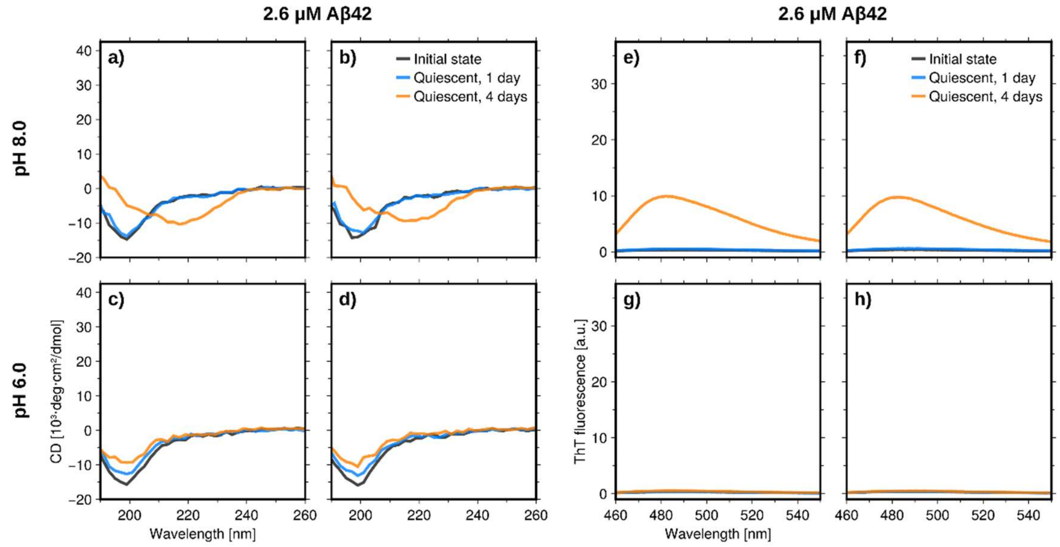

Figure S2. Kinetic experiments under quiescent conditions. The experiments were performed in the same way as the one presented in **Figure 1** of the main manuscript, except that aliquots of each sample were also measured with ThT fluorescence (200 μL sample + 50 μL 50 μM ThT in buffer, excitation at 440 nm). **a-d)** CD measurements. **e-h)** ThT fluorescence measurements of the same samples. The samples were prepared in duplicate.

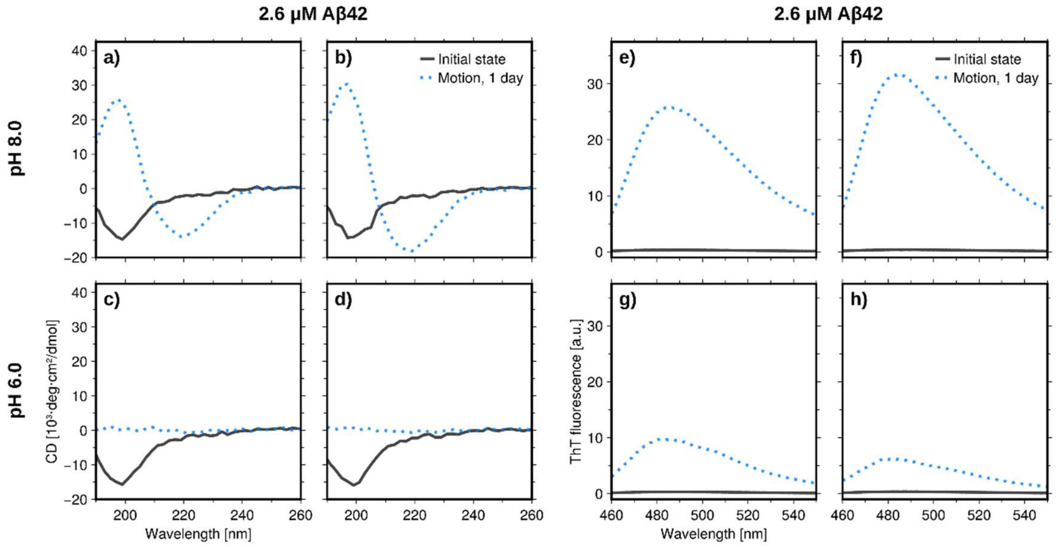

Figure S3. Kinetic experiments under rocking conditions. The experiments were performed in the same way as for **Figure S2**, except for the rocking motion (± 30° at 0.25 Hz, **Video S1**). **a-d)** CD measurements. **e-h)** ThT fluorescence measurements of the same samples. The samples were prepared in duplicate. All samples had aggregated after one day. The difference in fluorescence intensity between pH 8.0 and pH 6.0 persisted at the end-state (**Figure S4**).

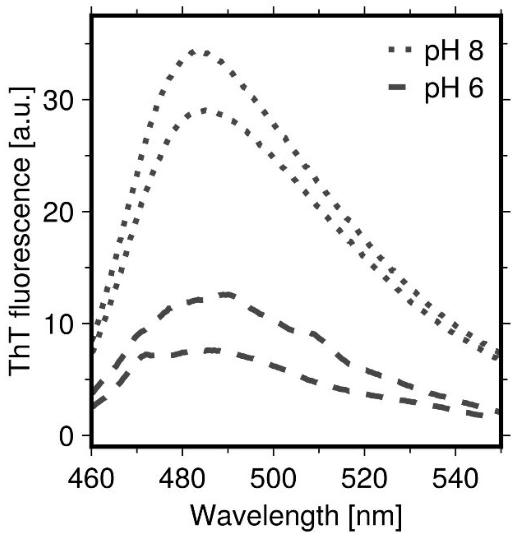

Figure S4. End-state ThT fluorescence spectra (the rocking-condition samples of **Figure S3** re-measured after a total of 1 day of rocking followed by 3 days of quiescence). The difference in fluorescence intensity persisted, indicating that it was due to the pH itself or the colloidal stability of the aggregates, rather than the completion degree of amyloid formation.

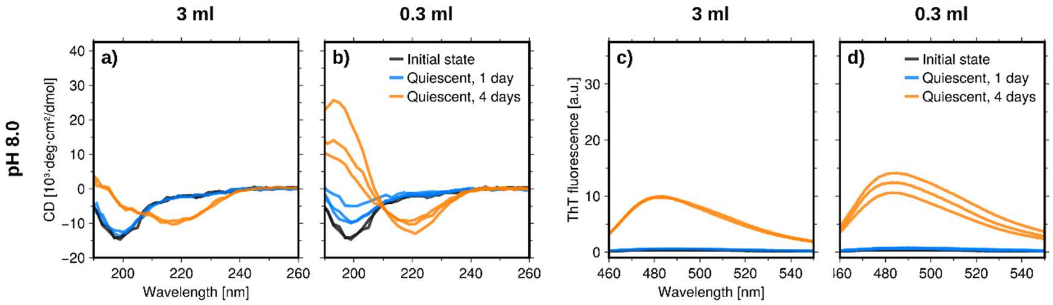

Figure S5. The effect of sample volume in the vials under quiescent conditions. **a** and **c** are the pH 8.0 samples from **Figure S2** replotted, whereas **b** and **d** are 0.3 ml samples in the same type of glass vials (low-volume samples). For the latter, each curve was a separate sample (rather than the same samples measured multiple times), but the initial state curves were the same as for the large-volume samples. The low-volume samples formed fibrils faster than the large-volume samples as judged by the CD spectra. The difference was less clear with ThT fluorescence. The reduction in CD amplitude relative to the initial state in **b** suggests loss of protein in the bulk (presumably due to surface adsorption) rather than partial aggregation, because at pH 8.0 partial aggregation is expected to change the shape of the spectrum.
