# Supplementary material for: Does amyloid fibril nucleation occur at surfaces only?
Source: Biophys J. 2025 Nov 6;125(1):29–34. doi: 10.1016/j.bpj.2025.11.002 (PMC12821009; doi:10.1016/j.bpj.2025.11.002)
Supplement: Document S2. Article plus supporting material [file mmc3.pdf]

Biophysical Journal
Biophysical letter

Biophysical Society

# Does amyloid fibril nucleation occur at surfaces only?

Jon Pallbo,[1,*] Sara Linse,[2] and Ulf Olsson[1]
[1]Physical Chemistry, Lund University, Lund, Sweden and [2]Biochemistry and Structural Biology, Lund University, Lund, Sweden

ABSTRACT The Aβ42 peptide (APP(672–713)), associated with Alzheimer disease, is highly prone to form amyloid fibrils and has been extensively studied through in vitro experiments. Such experiments represent a basis for understanding the biophysical chemistry of amyloid-related diseases. In this communication, we show that homogeneous primary nucleation in vitro of Aβ42 fibrils is a very rare event, implying that primary nucleation occurs almost exclusively at interfaces, by heterogeneous nucleation. Recognizing that the protein molecules in amyloid fibrils possess a two-dimensional fold, we discuss the nucleation in relation to protein folding and Levinthal's paradox. In the much more rapid heterogeneous nucleation, we suggest that one catalyzing effect is the significant reduction of the effective conformational space when a monomer polypeptide chain (strongly) adsorbs to a surface, facilitating its search for the target fold.

SIGNIFICANCE This work, dealing with primary nucleation of amyloid fibrils, has two main messages. The first is that changing the experimental setup in ways that one might at first assume to be insignificant, such as keeping samples in vials instead of multiwell plates, can have an order-of-magnitude effect on the results. This results from nucleation in the bulk being negligible relative to surface-induced amyloid formation. The second message is the proposed explanation for this observation. It is an alternative solution to Levinthal's paradox, which is applicable to amyloid formation at surfaces. It is highly relevant to consider these effects in research on amyloid formation because of the implications for experimental design and the interpretation of results.

Amyloid deposits in the brain are a hallmark of a number of neurodegenerative diseases, such as Alzheimer, Parkinson, and Huntington disease (1). The amyloids are ordered fibrillar protein/peptide aggregates, where identically 2D-folded protein molecules stack to form what is often referred to as a protofilament (2). The folds are truly 2D in the sense that the polypeptide chain does not cross itself in the fold. The 2D-folded protein contains a number of β-strands that participate in parallel intermolecular β-sheets propagating along the fibril with a characteristic periodicity of 4.7 Å. A filament typically contains one or two such protofilaments, and a fibril may in turn contain more than one filament that intertwines with a certain pitch length. Since the connection with neurodegenerative diseases was established, pathological amyloids have been characterized in vivo as well as in numerous in vitro studies (3).

An important characteristic property of amyloid proteins is their propensity to self-assemble into fibrils. Several methods can be used to monitor fibril formation (4). One common approach involves the use of noncovalent fluorescent probes such as thioflavin T (ThT) (5), which bind to amyloid fibrils with enhanced fluorescence, and multiwell plates to measure a range of samples simultaneously. By using sequence-homogeneous and ultrapure recombinant proteins, highly reproducible results on the formation kinetics can be obtained with such setups (6). This has allowed for testing kinetic models that include primary and secondary nucleation, fibril elongation and dissociation, and the possibility of fibril fragmentation (7,8).

It has been reported that the presence of surfaces, such as the water-air interface or the container/vial surface, may accelerate fibril formation by offering sites for heterogeneous nucleation (9–14). Surface effects have also been studied systematically by adding the interface in the form of colloidal particles to the solution (14,15).

The main aim of the present study was to characterize homogeneous amyloid nucleation in the bulk. Our strategy to reduce the possibility for heterogeneous nucleation was to

*Submitted September 5, 2025, and accepted for publication November 3, 2025.*
*Correspondence: jon.pallbo_arvidsson@fkem1.lu.se
Editor: Bernd Reif.
https://doi.org/10.1016/j.bpj.2025.11.002
© 2025 The Author(s). Published by Elsevier Inc. on behalf of Biophysical Society.
This is an open access article under the CC BY license (http://creativecommons.org/licenses/by/4.0/).

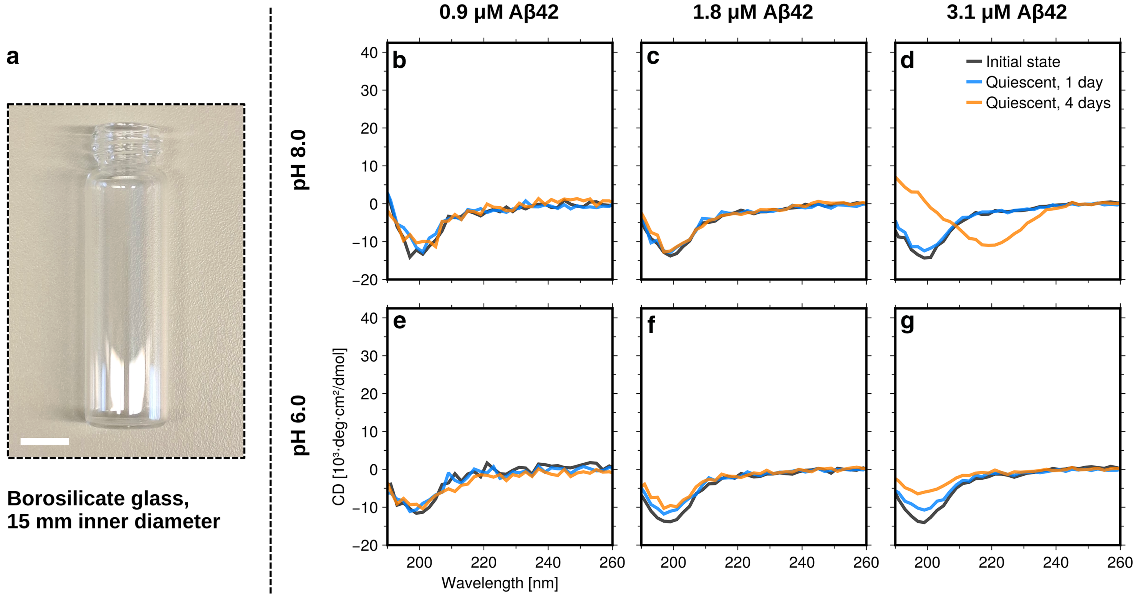


**FIGURE 1 Kinetics experiments under quiescent conditions. (*a*) Photograph of the kind of vial used in the experiments. The white scale bar corresponds to 10 mm. (*b–g*) CD spectra obtained immediately after sample preparation, and after 1 and 4 days of incubation at room temperature for a total of six separately prepared samples. Data at three different Aβ42 concentrations (0.9, 1.8, and 3.1 μM) and two different pH values (6.0 and 8.0) are compared.**

reduce the interface-to-volume ratio by using a large sample volume, approximately 3 mL, in borosilicate glass vials of 1.5 cm inner diameter (Fig. 1 *a*). We have used the amyloid-β protein in the form Aβ(M1-42), here abbreviated as Aβ42, which is associated with Alzheimer disease. Aβ42 is one of the most studied amyloid proteins in vitro (16). Expression and purification of Aβ42 followed a previously published protocol (17). Samples at three primary concentrations of Aβ42 were investigated, 0.9, 1.8 and 3.1 μM, and at two pH values, pH = 6.0 and pH = 8.0, in 20 mM sodium phosphate buffer with 0.2 mM EDTA. Additional experiments were performed with 2.0 and 2.6 μM Aβ42 (Figs. S1–S5). At the concentrations and pH values used, Aβ42 readily aggregates in PEGylated polystyrene multiwell plates, with the half time for fibril formation (defined as the time at which half the monomers have formed fibrils) being about 1–5 h (7) (Fig. S1).

To assess the formation of fibrils, we recorded circular dichroism (CD) spectra at certain time points over a total period of 4 days. Peptide concentrations were determined from the amplitude of the CD spectra at the initial state at pH = 8.0, using a conversion factor based on the normal light absorbance at 280 nm ($\varepsilon = 1440\ M^{-1}\ cm^{-1}$) and a CD spectrum for a reference Aβ42 sample under the same conditions. As a monomer, Aβ42 is essentially a random coil, showing a minimum in the CD spectrum at the wavelength $\lambda \approx 200$ nm. The β-sheet-rich amyloid fibrils, on the other hand, show a maximum in the CD spectrum at $\lambda \approx 200$ nm and a minimum at $\lambda \approx 220$ nm. For one set of samples, the vials were left standing at ambient temperature (20°C–23°C). The results are presented in Figs. 1 *b–g* and S2. For the two lowest concentrations in Fig. 1, all CD spectra are almost identical to the initial monomer state and consistent with a random coil structure. Thus, no aggregation is observed within 4 days of incubation under these quiescent conditions. For the highest concentration, no substantial changes in the spectra are observed after 1 day, just as for the other samples. However, after 4 days of incubation, there are some changes in the CD spectra. At pH = 8.0, a minimum at $\lambda \approx 220$ nm is observed, indicating the formation of some β-sheet-rich aggregates consistent with amyloid fibrils. At pH = 6.0, some amyloid formation might have also occurred after 4 days at the highest concentration since the signal is slightly different from the initial state. However, no qualitative change in the CD spectrum is observed (only the minimum at $\lambda \approx 200$ nm has become weaker) nor do we observe any increase in the ThT fluorescence in the 2.6-μM sample (Fig. S2). We attribute this to surface adsorption of protein monomers and/or aggregates on the glass vial wall, and possibly also at the water-air interface, as well as to absorption flattening due to formation of dense protein clusters (see below).

Agitation is known to accelerate fibril formation (13,18) by facilitating the detachment of nucleating species from interfaces (17,19). Hence, for a second set of samples, we let the vials undergo a rocking motion during the incubation (at 20°C–23°C), as illustrated in Fig. 2 *a*, with an amplitude of 30° and frequency of 0.25 Hz. The setup used to achieve the rocking motion is shown in Video S1. In this case, fibrils were formed within the first day. At pH = 8.0, the CD

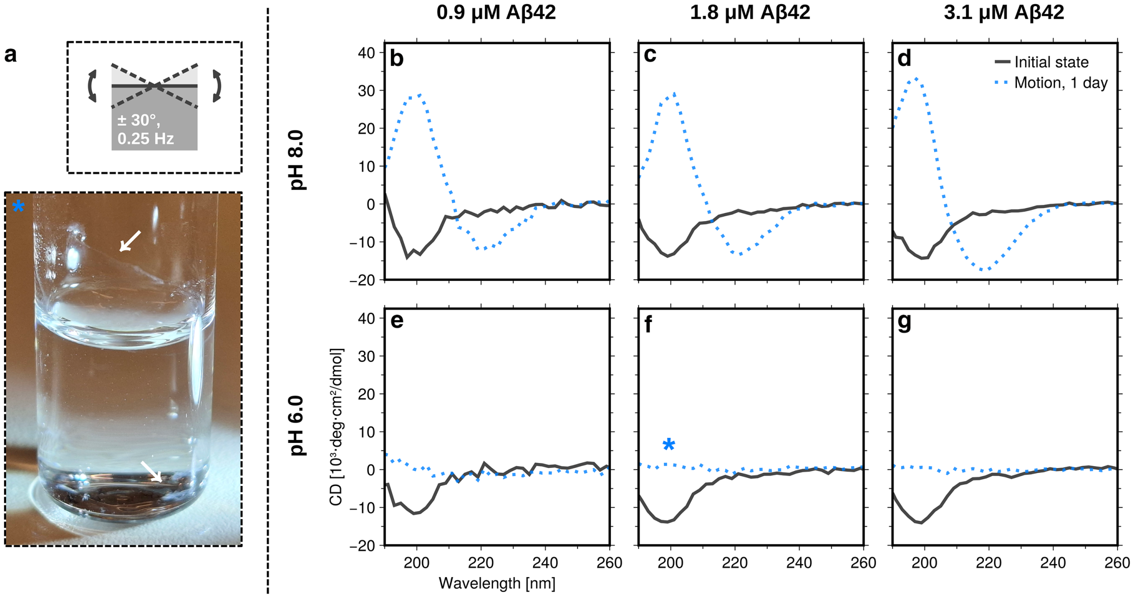


**FIGURE 2 Kinetics experiments under rocking conditions. (*a*) Top: schematic illustration of the rocking motion involved. Bottom: photograph of the 1.8-μM sample at pH = 6.0 after 1 day of rocking incubation, showing a macroscopic protein film on the vial wall in addition to sedimented precipitate at the bottom of the vial, as highlighted by white arrows. (*b–g*) CD spectra obtained immediately after sample preparation (same as in Fig. 1) and after 1 day of incubation, for a total of six separately prepared samples. Data at three different Aβ42 concentrations (0.9, 1.8, and 3.1 μM) and two different pH values (6.0 and 8.0) are compared.**

spectra (Figs. 2 *b–g* and S3) are characteristic of β-sheets and very similar to previously published spectra from Aβ42 fibrils (20). At pH = 6.0, no CD signal was obtained after incubation. However, aggregates had still formed (see ThT fluorescence in Fig. S3), but they were not colloidally stable. pH = 6.0 is close to the isoelectric point, and the fibrils form large clusters that stick to the glass vial wall or sediment to the bottom (Fig. 2 *a*). Formation of colloidally unstable clusters of Aβ42 fibrils has also been shown to occur at pH = 6.8 (21). Another contribution to the loss of CD signal at pH = 6.0 likely comes from absorption flattening due to a high density of the clusters (22), and there could also be an effect of light scattering.

The present results clearly show that under the solution conditions investigated here, homogeneous primary nucleation of Aβ42 fibrils is very slow and essentially does not occur at all. However, heterogeneous primary nucleation on surfaces, as well as secondary nucleation (new fibrils forming due to the presence of already existing fibrils), still occurs. In kinetic experiments on amyloid formation performed in medium volume multiwell plates the aggregation is much faster, and the primary nucleation event most likely involves heterogeneous nucleation at the container wall or at the sample-air interface (7,9,17).

The absence of homogeneous primary nucleation can possibly be understood if we recognize that the nucleation event involves protein folding. In the case of folding of globular proteins, Levinthal's paradox states that although a random search within the gigantic conformational space of a polypeptide chain for the correct fold would typically cover the age of the universe, proteins still fold on the microsecond to millisecond timescale (23,24). The so-called funnel hypothesis assumes pathways of the (far from random) folding process that involves folding intermediates, foldons, that form cooperatively with secondary structure that reflects the native state (25). This stepwise route to the native state involves the formation of intramolecular hydrogen bonds in the secondary structures that successively reduces the effective conformational space. The results of Monte Carlo simulations imply that no pathways need to be specified for fast folding to occur if the contacts within the native structure have on average longer lifetimes than nonnative ones (26).

In the amyloid fibril, the β-sheets are all intermolecular. There are β-strands, but no β-sheets, within the 2D-folded protein molecule. Although some side chains are partially buried between the β-strands, such flat structures would still be highly unstable for an isolated monomer in solution as large parts of these side chains would be exposed to water on both sides of the plane (Fig. 3). The fold is stabilized in the fibril where adjacent monomer planes serve to further bury the hydrophobic side chains. Thus, a possible explanation, at least partially, for the very slow homogeneous primary nucleation of amyloid fibrils is the very slow folding in the amyloid nucleation due to the few stabilizing intramolecular interactions within the fold.

The activation free energy of nucleation is often much smaller for heterogeneous primary nucleation on a surface compared with in the bulk. Heterogeneous nucleation is favored if the new phase wets the surface. A classic example

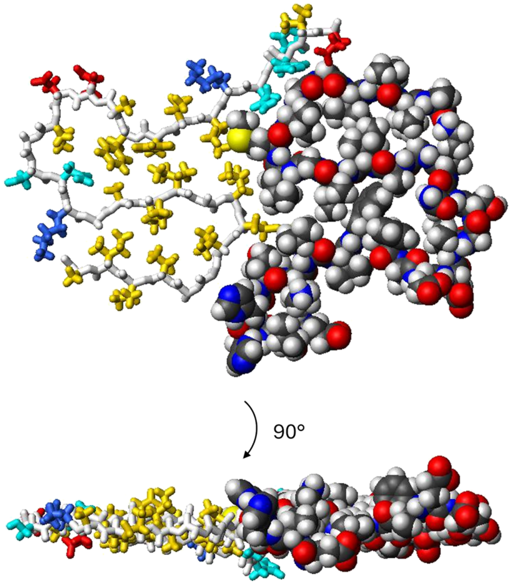


**FIGURE 3 The 2D fold of Aβ42 monomers within fibrils shown as a top view (*top*) and a side view (*bottom*) of two monomers in one plane of a filament. One monomer (*right*) is shown as a space-filling model using standard Corey–Pauling–Koltun (CPK) colors, and one monomer (*left*) is shown as a stick model with white backbone and side chains in yellow (hydrophobic), red (negatively charged), blue (positively charged), and cyan (hydrophilic). Two such filaments form an Aβ42 fibril with four monomers per plane. This figure was prepared from PDB: 5KK3 (including residues 12–42) using Molmol (27).**

is vapor condensation on a glass surface. Locally higher concentrations in the case of surface adsorption may also accelerate the process. A likely additional contribution to the much higher heterogeneous nucleation rate is that the adsorption to the interface significantly reduces the effective conformational space, speeding up the search for the target 2D fold. In the extreme case of a flat "pancake" adsorption, the conformational space is essentially reduced to 2D conformations only, which is a huge reduction compared with the conformational space in 3D. Similar ideas were invoked in a molecular dynamics simulation study of short peptides at a hydrophobic surface (11).

Amyloid nucleation of Aβ42 has been shown to be a two-step process (28), analogous to molecular crystallization (29). The first step involves the formation of clusters, oligomers, which in a second step are converted to the fibril state that grows through elongation. Compared with fibrils, the oligomers are relatively unstable, of low growth rate, and display a different structure (30). Oligomers dissociate more rapidly than they convert (28), and the second step involves a massive structural rearrangement leading to the fibril state. In the case of heterogeneous nucleation, the first step, per definition, takes place at the surface and it is likely that so also does the second step. The folding rearrangement (conversion) may indeed be the rate-determining step of the nucleation process, which is significantly facilitated at the surface.

Interestingly, rocking the sample vials lead to fibril formation within 1 day (Figs. 2 and S3). The reasons for this can likely be traced to the interfaces, but a detailed analysis is nontrivial and beyond the scope of the present communication. Increasing the area-to-volume ratio by using low-volume samples in the same type of glass vials promotes faster aggregation (Fig. S5), but it is still much slower than typically observed in multiwell plates. This highlights the fact that the type of surface or the combination of surfaces with motion are important (13). Tilting the sample by 30° expands the water-air interface by approximately 15%. Thus, the rocking leads to a periodic expansion-contraction of this interface. There is also the periodic wetting-dewetting of the glass vial wall (31). Furthermore, diffusion in the bulk limits mass transfer to the surface in the quiescent condition and might contribute to a lag phase (32), whereas rocking the sample likely enhances the mass transfer. Similar acceleration effects were recently analyzed in a systematic study of the effects of plate reader motion, start/stop, in high-throughput kinetics assays (17). It was concluded that the shear forces caused by plate motion accelerated both primary (presumably heterogeneous) and secondary nucleation of the fibril formation pathway.

To summarize, we have shown that homogeneous primary nucleation of Aβ42 fibrils in vitro is a very rare event, possibly not occurring at any perceivable rate. This implies that primary nucleation is heterogeneous and, importantly, that subtle changes in the experimental setup can have orders-of-magnitude effects on the observed amyloid formation kinetics. As one contributing factor, we suggest that in solution the search in conformational space for the correct amyloid fold essentially is random, in contrast to that of globular proteins, because of relatively few intramolecular interactions in the monomer of the fibril state. This is a general and nonspecific argument, and we hypothesize that it should be applicable also for other amyloid systems. In fact, it is known that α-synuclein does not aggregate within typical experimental time frames under quiescent conditions in vitro (33), but it requires the presence of "catalytic" surfaces in the form of anionic lipid bilayers (34,35), surfactant micelles (36), polystyrene surfaces (37), or the use of high-frequency vibrations (38).

## DATA AVAILABILITY

The data are available at https://github.com/saralinse/Published_Data/tree/BPJ_2025_surface_nucleation.

## ACKNOWLEDGMENTS

This work was supported by the European Research Council (AdG 101097824 to S.L.), the Swedish Research Council (2015-00143 to S.L. and 2020-04633 to U.O.), and Knut och Alice Wallenberg's Foundation (2022.0059 to S.L. and U.O.).

## AUTHOR CONTRIBUTIONS

U.O., J.P., and S.L. conceptualized and designed the research. J.P. performed the experiments and analyzed the data. U.O., J.P., and S.L. wrote and edited the manuscript. S.L. provided protein material. U.O. and S.L. acquired funding for the research.

## DECLARATION OF INTERESTS

The authors declare no competing interests.

## SUPPORTING MATERIAL

Supporting material can be found online at https://doi.org/10.1016/j.bpj.2025.11.002.

## REFERENCES


1. Chiti, F., and C. M. Dobson. 2017. Protein Misfolding, Amyloid Formation, and Human Disease: A Summary of Progress Over the Last Decade. *Annu. Rev. Biochem.* 86:27–68. https://doi.org/10.1146/annurev-biochem-061516-045115.
2. Eisenberg, D. S., and M. R. Sawaya. 2017. Structural Studies of Amyloid Proteins at the Molecular Level. *Annu. Rev. Biochem.* 86:69–95. https://doi.org/10.1146/annurev-biochem-061516-045104.
3. Ke, P. C., R. Zhou, …, R. Mezzenga. 2020. Half a Century of Amyloids: Past, Present and Future. *Chem. Soc. Rev.* 49:5473–5509. https://doi.org/10.1039/c9cs00199a.
4. Arosio, P., T. P. J. Knowles, and S. Linse. 2015. On the Lag Phase in Amyloid Fibril Formation. *Phys. Chem. Chem. Phys.* 17:7606–7618. https://doi.org/10.1039/C4CP05563B.
5. Levine, H. 1993. Thioflavine T Interaction with Synthetic Alzheimer's Disease β -amyloid Peptides: Detection of Amyloid Aggregation in Solution. *Protein Sci.* 2:404–410. https://doi.org/10.1002/pro.5560020312.
6. Hellstrand, E., B. Boland, …, S. Linse. 2010. Amyloid β-Protein Aggregation Produces Highly Reproducible Kinetic Data and Occurs by a Two-Phase Process. *ACS Chem. Neurosci.* 1:13–18. https://doi.org/10.1021/cn900015v.
7. Cohen, S. I. A., S. Linse, …, T. P. J. Knowles. 2013. Proliferation of Amyloid-β42 Aggregates Occurs through a Secondary Nucleation Mechanism. *Proc. Natl. Acad. Sci. USA.* 110:9758–9763. https://doi.org/10.1073/pnas.1218402110.
8. Dear, A. J., G. Meisl, …, T. P. J. Knowles. 2020. The Catalytic Nature of Protein Aggregation. *J. Chem. Phys.* 152:045101. https://doi.org/10.1063/1.5133635.
9. Campioni, S., G. Carret, …, R. Riek. 2014. The Presence of an Air–Water Interface Affects Formation and Elongation of α Synuclein Fibrils. *J. Am. Chem. Soc.* 136:2866–2875. https://doi.org/10.1021/ja412105t.
10. Zhu, M., P. O. Souillac, …, A. L. Fink. 2002. Surface-Catalyzed Amyloid Fibril Formation. *J. Biol. Chem.* 277:50914–50922. https://doi.org/10.1074/jbc.m207225200.
11. Nikolic, A., S. Baud, …, R. Pomès. 2011. Molecular Mechanism of β-sheet Self-organization at Water-hydrophobic Interfaces. *Proteins.* 79:1–22. https://doi.org/10.1002/prot.22854.
12. Grigolato, F., and P. Arosio. 2021. The Role of Surfaces on Amyloid Formation. *Biophys. Chem.* 270:106533. https://doi.org/10.1016/j.bpc.2020.106533.
13. Morinaga, A., K. Hasegawa, …, H. Naiki. 2010. Critical Role of Interfaces and Agitation on the Nucleation of Aβ Amyloid Fibrils at Low Concentrations of Aβ Monomers. *Biochim. Biophys. Acta.* 1804:986–995. https://doi.org/10.1016/j.bbapap.2010.01.012.
14. Vácha, R., S. Linse, and M. Lund. 2014. Surface Effects on Aggregation Kinetics of Amyloidogenic Peptides. *J. Am. Chem. Soc.* 136:11776–11782. https://doi.org/10.1021/ja505502e.
15. Linse, S., C. Cabaleiro-Lago, …, K. A. Dawson. 2007. Nucleation of Protein Fibrillation by Nanoparticles. *Proc. Natl. Acad. Sci. USA.* 104:8691–8696. https://doi.org/10.1073/pnas.0701250104.
16. Hamley, I. W. 2012. The Amyloid Beta Peptide: A Chemist's Perspective. Role in Alzheimer's and Fibrillization. *Chem. Rev.* 112:5147–5192. https://doi.org/10.1021/cr3000994.
17. Axell, E., J. Hu, …, S. Linse. 2024. The Role of Shear Forces in Primary and Secondary Nucleation of Amyloid Fibrils. *Proc. Natl. Acad. Sci. USA.* 121:e2322572121. https://doi.org/10.1073/pnas.2322572121.
18. Nielsen, L., R. Khurana, …, A. L. Fink. 2001. Effect of Environmental Factors on the Kinetics of Insulin Fibril Formation: Elucidation of the Molecular Mechanism. *Biochemistry.* 40:6036–6046. https://doi.org/10.1021/bi002555c.
19. Xu, C. K., G. Meisl, …, T. P. J. Knowles. 2024. α-Synuclein Oligomers Form by Secondary Nucleation. *Nat. Commun.* 15:7083. https://doi.org/10.1038/s41467-024-50692-4.
20. Marshall, K. E., D. M. Vadukul, …, L. C. Serpell. 2016. A Critical Role for the Self-Assembly of Amyloid-β1-42 in Neurodegeneration. *Sci. Rep.* 6:30182. https://doi.org/10.1038/srep30182.
21. Törnquist, M., R. Cukalevski, …, S. Linse. 2020. Ultrastructural Evidence for Self-Replication of Alzheimer-Associated Aβ42 Amyloid along the Sides of Fibrils. *Proc. Natl. Acad. Sci. USA.* 117:11265–11273. https://doi.org/10.1073/pnas.1918481117.
22. Wittung, P., J. Kajanus, …, B. G. Malmström. 1994. Absorption Flattening in the Optical Spectra of Liposome-entrapped Substances. *FEBS Lett.* 352:37–40. https://doi.org/10.1016/0014-5793(94)00912-0.
23. Levinthal, C. 1969. How to Fold Graciously. *Mössbaun Spectroscopy in Biological Systems Proceedings Univ. of Illinois Bulletin.* 67:22–24.
24. Dill, K. A., and J. L. MacCallum. 2012. The Protein-Folding Problem, 50 Years On. *Science.* 338:1042–1046. https://doi.org/10.1126/science.1219021.
25. Englander, S. W., and L. Mayne. 2014. The Nature of Protein Folding Pathways. *Proc. Natl. Acad. Sci. USA.* 111:15873–15880. https://doi.org/10.1073/pnas.1411798111.
26. Linse, S., and B. Linse. 2007. Protein Folding through Kinetic Discrimination. *J. Am. Chem. Soc.* 129:8481–8486. https://doi.org/10.1021/ja070386e.
27. Koradi, R., M. Billeter, and K. Wüthrich. 1996. MOLMOL: A Program for Display and Analysis of Macromolecular Structures. *J. Mol. Graph.* 14:51–55. https://doi.org/10.1016/0263-7855(96)00009-4.
28. Michaels, T. C. T., A. Šarić, …, T. P. J. Knowles. 2020. Dynamics of Oligomer Populations Formed during the Aggregation of Alzheimer's Aβ42 Peptide. *Nat. Chem.* 12:445–451. https://doi.org/10.1038/s41557-020-0452-1.
29. Vekilov, P. G. 2010. The Two-Step Mechanism of Nucleation of Crystals in Solution. *Nanoscale.* 2:2346–2357. https://doi.org/10.1039/c0nr00628a.
30. Lendel, C., M. Bjerring, …, T. Härd. 2014. A Hexameric Peptide Barrel as Building Block of Amyloid-β Protofibrils. *Angew. Chem. Int. Ed.* 53:12756–12760. https://doi.org/10.1002/anie.201406357.
31. Chouchane, K., T. Frachon, …, M. Weidenhaupt. 2022. Insulin Aggregation Starts at Dynamic Triple Interfaces, Originating from Solution Agitation. *Colloids Surf., B.* 214:112451. https://doi.org/10.1016/j.colsurfb.2022.112451.
32. Pallbo, J., M. Fornasier, …, U. Olsson. 2025. Air–Water Interfacial Adsorption of the Chaperone Protein DNAJB6b. *Langmuir.* 41:19146–19155. https://doi.org/10.1021/acs.langmuir.5c01237.
33. Horne, R. I., M. A. Metrick, …, M. Vendruscolo. 2023. Secondary Processes Dominate the Quiescent, Spontaneous Aggregation of α Synuclein at Physiological pH with Sodium Salts. *ACS Chem. Neurosci.* 14:3125–3131. https://doi.org/10.1021/acschemneuro.3c00282.

34. Galvagnion, C., A. K. Buell, …, C. M. Dobson. 2015. Lipid Vesicles Trigger α-Synuclein Aggregation by Stimulating Primary Nucleation. *Nat. Chem. Biol.* 11:229–234. https://doi.org/10.1038/nchembio.1750.

35. Stevenson, A., R. Staats, …, T. C. T. Michaels. 2025. Global Kinetic Model of Lipid-Induced α-Synuclein Aggregation and Its Inhibition by Small Molecules. *Proc. Natl. Acad. Sci. USA.* 122:e2422427122. https://doi.org/10.1073/pnas.2422427122.

36. Giehm, L., C. L. P. Oliveira, …, D. E. Otzen. 2010. SDS-Induced Fibrillation of α-Synuclein: An Alternative Fibrillation Pathway. *J. Mol. Biol.* 401:115–133. https://doi.org/10.1016/j.jmb.2010.05.060.

37. Grey, M., S. Linse, …, E. Sparr. 2011. Membrane interaction of α-synuclein in different aggregation states. *J. Parkinsons Dis.* 1:359–371. https://doi.org/10.3233/JPD-2011-11067.

38. Farzadfard, A., T. O. Mason, …, A. K. Buell. 2025. The Amplification of Alpha-Synuclein Amyloid Fibrils Is Suppressed under Fully Quiescent Conditions. *Angew. Chem. Int. Ed.* 64:e202419173. https://doi.org/10.1002/anie.202419173.

Biophysical Journal, Volume *125*

## Supplemental information

# Does amyloid fibril nucleation occur at surfaces only?

**Jon Pallbo, Sara Linse, and Ulf Olsson**

# Supporting information for:

# Does amyloid fibril nucleation occur at surfaces only?

Jon Pallbo[1]*, Sara Linse[2], and Ulf Olsson[1]

[1]Physical Chemistry, Lund University, P.O. Box 124, 221 00 Lund, Sweden

[2]Biochemistry and Structural Biology, Lund University, P.O. Box 124, 221 00 Lund, Sweden

*jon.pallbo_arvidsson@fkem1.lu.se

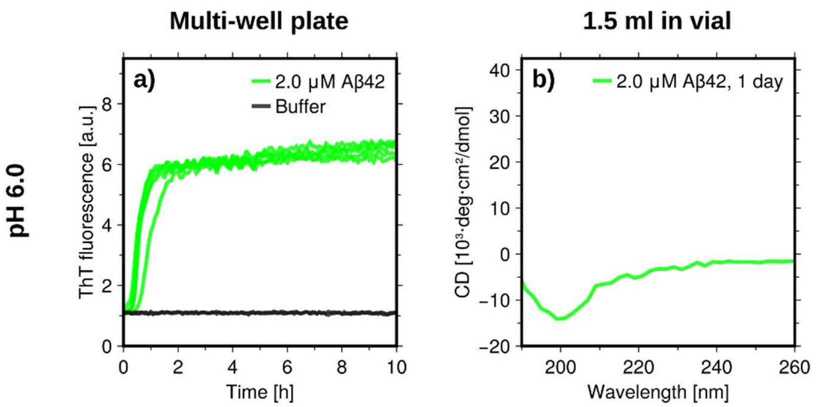


**Figure S1.** Comparison between amyloid formation in **a)** a multi-well plate (Corning 3881, 96-well half area) and **b)** a vial (borosilicate glass, 13 mm inner diameter). The same Aβ42 monomer solution (2.0 µM) was used to prepare all samples. The multi-well plate samples had aggregated within 2 hours, whereas no sign of aggregation was seen for the sample in the vial after one day of quiescent incubation. The buffer was the same as for the other experiments (at pH 6.0) with the addition of 9 µM Thioflavin T (ThT) in all samples.

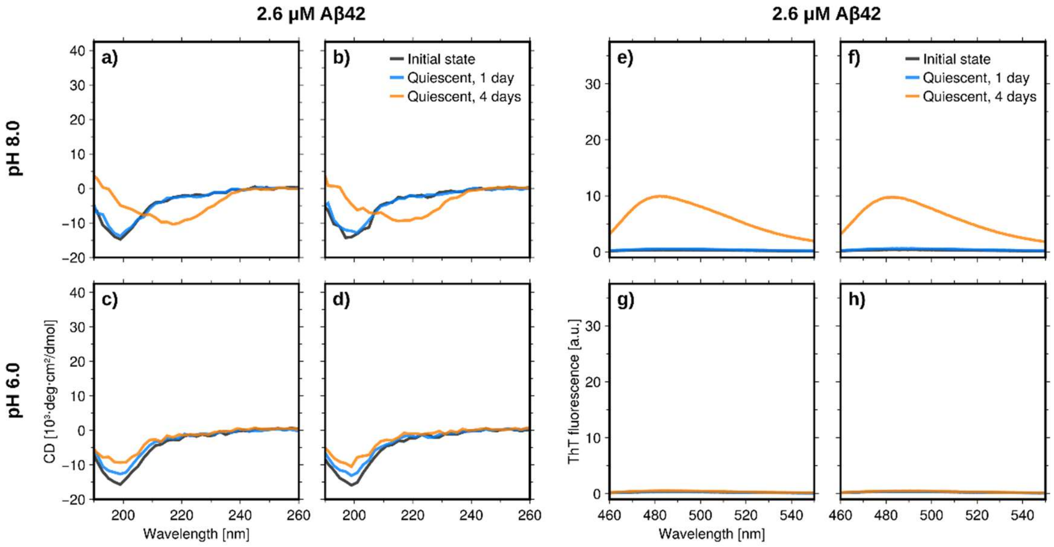


**Figure S2.** Kinetic experiments under quiescent conditions. The experiments were performed in the same way as the one presented in **Figure 1** of the main manuscript, except that aliquots of each sample were also measured with ThT fluorescence (200 µL sample + 50 µL 50 µM ThT in buffer, excitation at 440 nm). **a-d)** CD measurements. **e-h)** ThT fluorescence measurements of the same samples. The samples were prepared in duplicate.

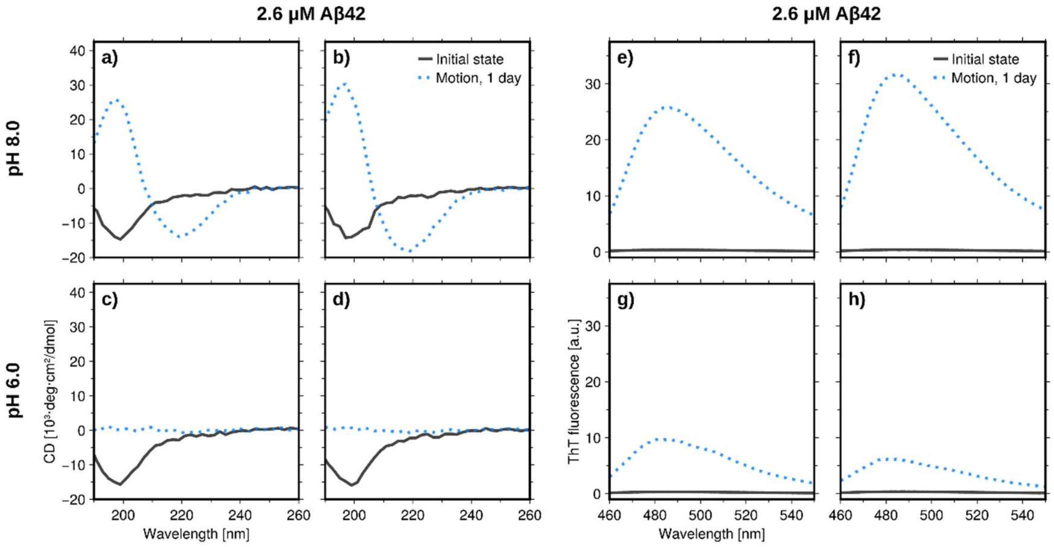


**Figure S3.** Kinetic experiments under rocking conditions. The experiments were performed in the same way as for **Figure S2**, except for the rocking motion (± 30° at 0.25 Hz, **Video S1**). **a-d)** CD measurements. **e-h)** ThT fluorescence measurements of the same samples. The samples were prepared in duplicate. All samples had aggregated after one day. The difference in fluorescence intensity between pH 8.0 and pH 6.0 persisted at the end-state (**Figure S4**).

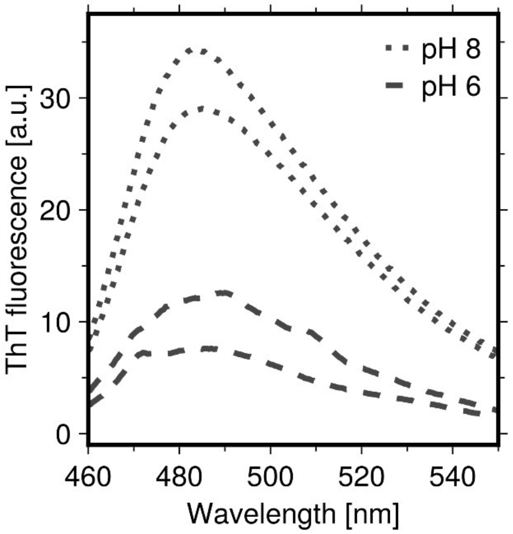


**Figure S4.** End-state ThT fluorescence spectra (the rocking-condition samples of **Figure S3** re-measured after a total of 1 day of rocking followed by 3 days of quiescence). The difference in fluorescence intensity persisted, indicating that it was due to the pH itself or the colloidal stability of the aggregates, rather than the completion degree of amyloid formation.

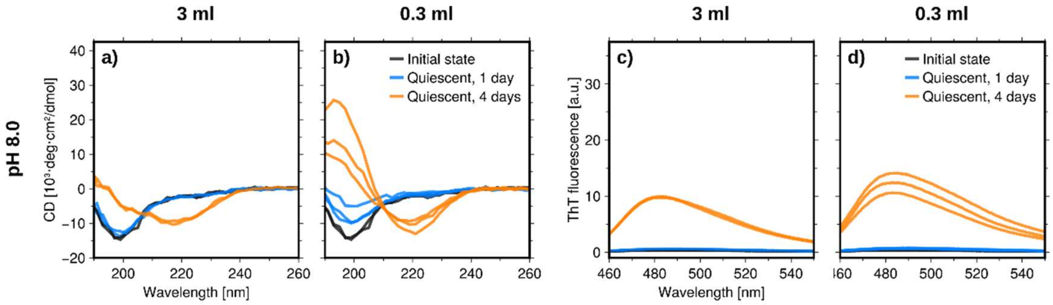


**Figure S5.** The effect of sample volume in the vials under quiescent conditions. **a** and **c** are the pH 8.0 samples from **Figure S2** replotted, whereas **b** and **d** are 0.3 ml samples in the same type of glass vials (low-volume samples). For the latter, each curve was a separate sample (rather than the same samples measured multiple times), but the initial state curves were the same as for the large-volume samples. The low-volume samples formed fibrils faster than the large-volume samples as judged by the CD spectra. The difference was less clear with ThT fluorescence. The reduction in CD amplitude relative to the initial state in **b** suggests loss of protein in the bulk (presumably due to surface adsorption) rather than partial aggregation, because at pH 8.0 partial aggregation is expected to change the shape of the spectrum.
